# Unexpected finding in an adult with ventricular fibrillation and an accessory pathway: non-compaction cardiomyopathy

**DOI:** 10.1007/s12471-013-0513-9

**Published:** 2014-01-08

**Authors:** A. Yaksh, D. Haitsma, T. Ramdjan, K. Caliskan, T. Szili-Torok, N. M. S. de Groot

**Affiliations:** 1 Department of Cardiology, Thoraxcentrum - Ba 579, Erasmus MC, ‘s Gravendijkwal 230, 3015 CE Rotterdam, the Netherlands; 2 Department of Cardiology, Thoraxcentrum - Ba 577, Erasmus MC, ‘s Gravendijkwal 230, 3015 CE Rotterdam, the Netherlands; 3 Department of Cardiology, Erasmus Medical Center, Rotterdam, the Netherlands

## Introduction

In this report, we demonstrate a patient presenting with an out-of-hospital cardiac arrest due to ventricular fibrillation (VF). At the hospital the presence of an accessory pathway could be seen on the surface electrocardiogram (ECG). Surprisingly, cardiac imaging also showed the presence of isolated left ventricular non-compaction cardiomyopathy (INVM).

INVM was first described in 1984 by Engberding et al. as an unclassified cardiomyopathy [1]. It is assumed to be the result of an arrest of the compaction process during the normal development of the heart (week 5–8). In INVM, the spaces within the intertrabeculated meshwork persist with deep recesses and no other cardiac abnormalities [1, 2]. Clinical presentation of INVM includes heart failure, thromboembolic events and arrhythmias [1, 3, 5, 7]. Conduction abnormalities and arrhythmias observed in INVM patients are left or right bundle branch block, supraventricular tachycardia and ventricular tachycardia [1–3, 5–10].

However, the presence of an accessory pathway and INVM in one patient with VF has never been described before.

## Case report

**Fig. 1:**
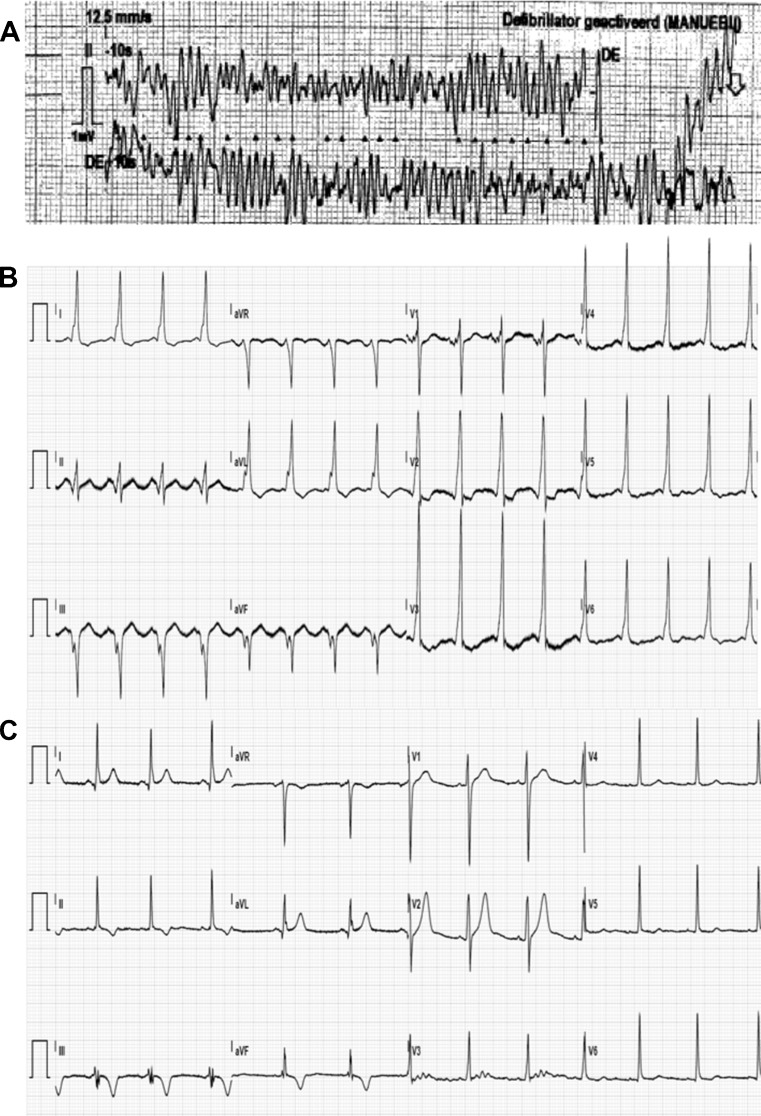
Electrocardiograms. **a** Documentation of ventricular fibrillation by ambulance paramedics. **b** ECG recorded on arrival to the emergency department, demonstrating sinus rhythm 99 beats/min, left axis, PR interval 115 ms (pre-excitation), QRS duration 144 ms, QTc 457 ms, delta waves (positive in I, aVL, V1-6; negative in II, III, aVF). **c** ECG after ablation of the accessory pathway, demonstrating sinus rhythm 70 beats/min, PR interval 122 ms, QRS duration 96 ms, QTc 391 ms, no delta waves, T-wave inversion in the inferior leads, left ventricular hypertrophy with associated depolarisation disorders

**Fig. 2:**
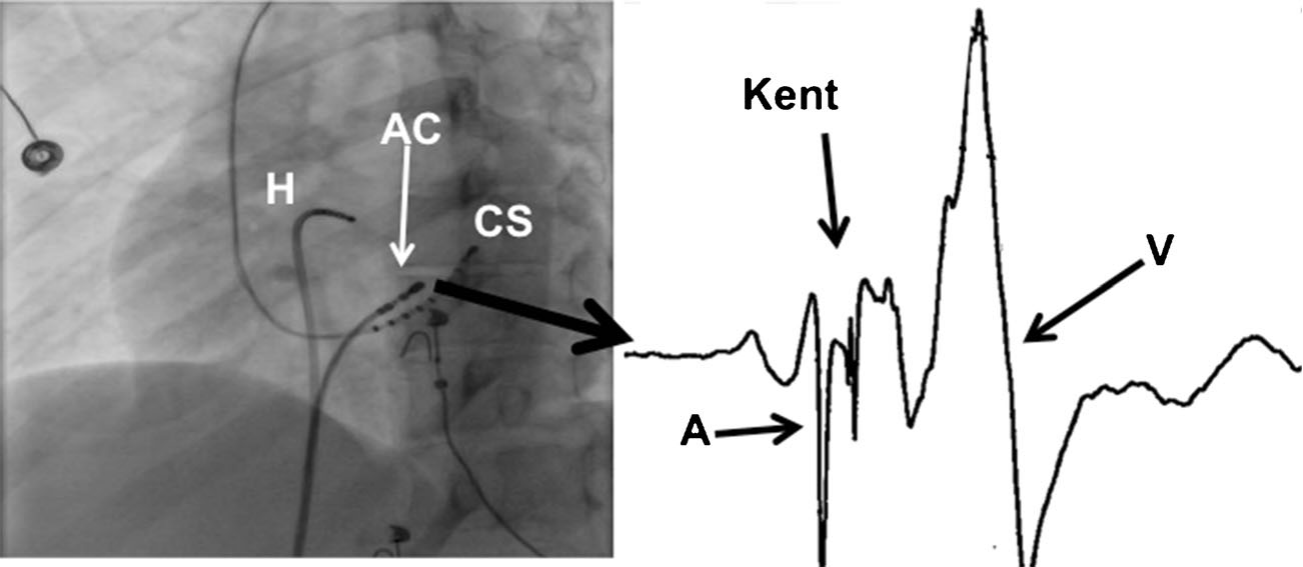
Catheter Ablation. *Right panel*: fluoroscopic image in LAO during the electrophysiology procedure. The ablation catheter (AC) located at the right-sided posteroseptal accessory pathway, a multipolar catheter in the coronary sinus (CS) and a quadripolar catheter on the His bundle (H). *Left panel*: the bipolar electrogram recorded from the ablation catheter located at the right-sided posteroseptal accessory pathway.

**Fig. 3:**
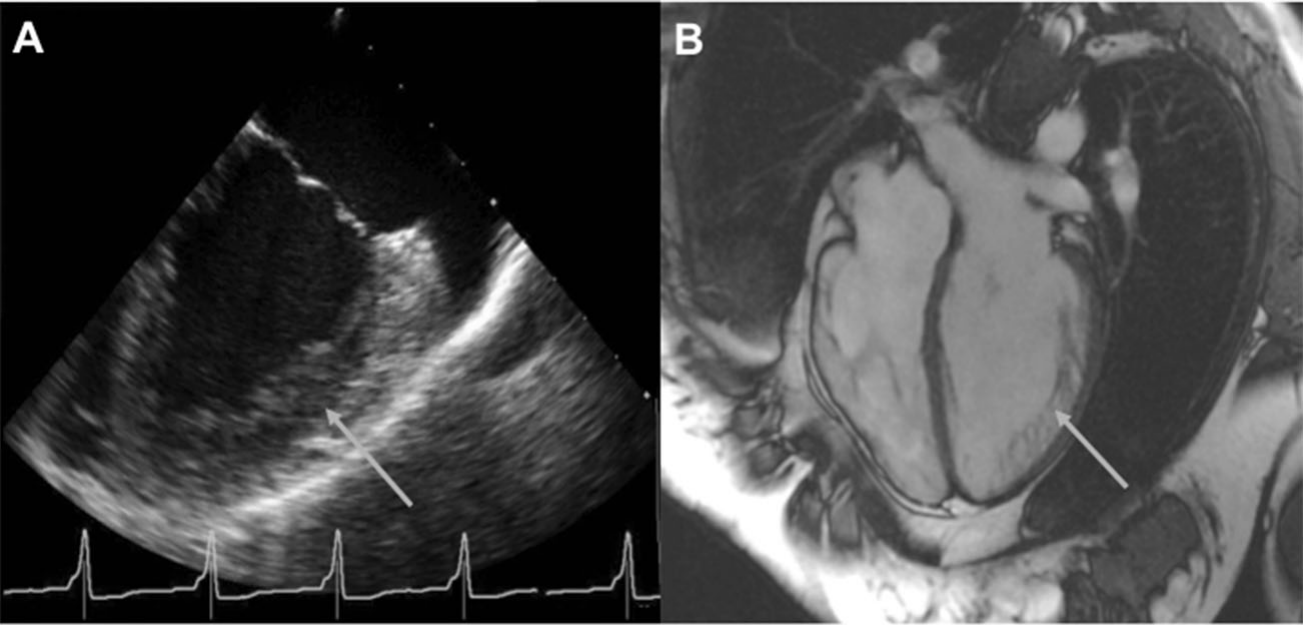
Cardiac imaging. Echocardiogram (**a**) and a magnetic resonance image (**b**) showing hypertrabeculation of the left ventricle (*arrow*)

A 19-year-old female presented to the emergency department after an out-of-hospital cardiac arrest due to VF. After alcohol consumption she jumped off a 1 m high pier into the water. While dressing she complained of dizziness, palpitations and breathlessness. She collapsed near her car and lost consciousness. The paramedics arrived within 7 min and provided cardiopulmonary resuscitation. VF was documented on arrival (Fig. 1). After three DC shocks sinus rhythm resumed and due to a low Glasgow Coma Score she was intubated. At the intensive cardiac care unit therapeutic hypothermia was induced for 24 h. She regained consciousness without any signs of persistent neurological injury. Anamnestic there were no previous palpitations or (near) collapses. The patient had noted that she was relatively quickly exhausted during physical exercise. Despite this, she played field hockey without any restraints. Her family history was negative for cardiovascular diseases, arrhythmias or sudden cardiac death. The 12-lead ECG after defibrillation showed pre-excitation with delta waves (positive in I, aVL, V1-6; negative in II, III, aVF) suggestive of a right-sided posteroseptal accessory pathway (Fig. 1). Therefore, during hospitalisation, the patient underwent an electrophysiology study. Figure 2 shows a Kent potential recorded at a right-sided posteroseptal bypass tract. The accessory pathway was successfully ablated at this site. The surface ECG after the ablation procedure showed no pre-excitation (PR 122 ms) and no delta waves (Fig. 1). Hence, VF was most likely due to the presence of an accessory pathway. The only abnormality on the surface ECG was left ventricular hypertrophy associated with depolarisation disorders. Transthoracic echocardiography showed normal left ventricular function, no significant valvular dysfunction and normal atrial dimensions. However, the left ventricle was hypertrabeculated and dilated (diastole 56 mm, systole 38 mm), suspicious for non-compaction cardiomyopathy. The diagnosis of non-compaction cardiomyopathy was confirmed by magnetic resonance imaging (MRI) (Fig. 3). Based on this finding, a subcutaneous ICD was implanted for secondary prevention in this 19-year-old patient.

## Discussion

The prevalence of INVM differs between 0.014 % (adult series) and 0.14 % in paediatric series [3]. Reported differences in the prevalence of INVM are probably caused by increased awareness of the existence of the disease over time. The true prevalence may be even higher, because only symptomatic patients are screened for INVM [2]. Echocardiography is useful for diagnosing INVM but recently MRI has proven to be more accurate for diagnosing INVM [1, 4–6]. The time course of development of ventricular tachyarrhythmia in INVM patients is at present unknown but an ICD is recommended for primary prevention of ventricular tachyarrhythmia [1–3, 6–10]

So far, an accessory pathway has only been described in two adult and four paediatric INVM patients. However, an adolescent patient with a Wolff-Parkinson-White syndrome and INVM presenting with VF has to our knowledge never been described before. Based on clinical data, it is impossible to determine whether VF was the result of either the INVM or atrial fibrillation with fast conduction over the accessory pathway.

In conclusion, we describe a 19-year-old patient who presented with an out-of-hospital cardiac arrest due to VF in the presence of a right-sided posteroseptal located accessory pathway. Surprisingly, we also found an INVM.

## References

[1] Engberding R, Yelbuz T, Breithardt G (2007). Isolated noncompaction of the left ventricular myocardium – a review of the literature two decades after the initial case description. Clin Res Cardiol.

[2] Chin TK, Perloff JK, Williams RG (1990). Isolated noncompaction of left ventricular myocardium. A study of eight cases. Circulation.

[3] Aras D, Tufekcioglu O, Ergun K (2006). Clinical features of isolated ventricular noncompaction in adults long-term clinical course, echocardiographic properties, and predictors of left ventricular failure. J Card Fail.

[4] De Groot-de Laat LE, Krenning BJ, ten Cate FJ (2005). Usefulness of contrast echocardiography for diagnosis of left ventricular noncompaction. Am J Cardiol.

[5] Lofiego C, Biagini E, Pasquale F (2007). Wide spectrum of presentation and variable outcomes of isolated left ventricular non-compaction. Heart.

[6] Sengupta PP, Mohan JC, Mehta V (2004). Comparison of echocardiographic features of noncompaction of the left ventricle in adults versus idiopathic dilated cardiomyopathy in adults. Am J Cardiol.

[7] Oechslin EN, Attenhofer Jost CH, Rojas JR (2000). Long-term follow-up of 34 adults with isolated left ventricular noncompaction: a distinct cardiomyopathy with poor prognosis. J Am Coll Cardiol.

[8] Ichida F, Hamamichi Y, Miyawaki T (1999). Clinical features of isolated noncompaction of the ventricular myocardium: long-term clinical course, hemodynamic properties, and genetic background. J Am Coll Cardiol.

[9] Murphy RT, Thaman R, Blanes JG (2005). Natural history and familial characteristics of isolated left ventricular non-compaction. Eur Heart J.

[10] Stöllberger C, Finsterer J, Blazek G (2002). Left ventricular hypertrabeculation/noncompaction and association with additional cardiac abnormalities and neuromuscular disorders. Am J Cardiol.

